# Opposite Roles of Tra2β and SRSF9 in the v10 Exon Splicing of CD44

**DOI:** 10.3390/cancers12113195

**Published:** 2020-10-30

**Authors:** Jagyeong Oh, Yongchao Liu, Namjeong Choi, Jiyeon Ha, Davide Pradella, Claudia Ghigna, Xuexiu Zheng, Haihong Shen

**Affiliations:** 1 School of life Sciences, Gwangju Institute of Science and Technology, Gwangju 500-712, Korea; jgoh@gist.ac.kr (J.O.); yongchao@gist.ac.kr (Y.L.); njchoi@gist.ac.kr (N.C.); hajiyn@gist.ac.kr (J.H.); 2 Istituto di Genetica Molecolare Luigi Luca Cavalli Sforza-Consiglio Nazionale delle Ricerche Via Abbiategrasso 207, 27100 Pavia, Italy; davide.pradella@igm.cnr.it (D.P.); arneri@igm.cnr.it (C.G.)

**Keywords:** alternative splicing, Tra2β, SRSF9, CD44, v10

## Abstract

**Simple Summary:**

Alternative splicing is one of the most regulated processes of eukaryotic gene expression. From one gene, multiple mRNA isoforms are produced by alternative splicing decision. Alternative splicing of the 9 variable exons of human CD44 pre-mRNA produces various mRNAs that are involved in different aspects of cancer progression and development. Here we identify Tra2β and SRSF9 as regulatory proteins with opposite roles in CD44 exon v10 splicing. While Tra2β promotes v10 inclusion through interacting with GAAGACG sequence in v10, SRSF9 inhibits v10 inclusion by binding to AAGAC. Our results provide a novel insight into the regulation of CD44 splicing.

**Abstract:**

CD44 is a transmembrane glycoprotein involved in cell–cell and cell–matrix interactions. Several CD44 protein isoforms are generated in human through alternative splicing regulation of nine variable exons encoding for the extracellular juxta-membrane region. While the CD44 splicing variants have been described to be involved in cancer progression and development, the regulatory mechanism(s) underlying their production remain unclear. Here, we identify Tra2β and SRSF9 as proteins with opposite roles in regulating CD44 exon v10 splicing. While Tra2β promotes v10 inclusion, SRSF9 inhibits its inclusion. Mechanistically, we found that both proteins are able to target v10 exon, with GAAGAAG sequence being the binding site for Tra2β and AAGAC that for SRSF9. Collectively, our data add a novel layer of complexity to the sequential series of events involved in the regulation of CD44 splicing.

## 1. Introduction

Pre-mRNA splicing is a molecular process by which a newly made precursor messenger RNA (pre-mRNA) transcript is transformed into a mature messenger RNA (mRNA). In this maturation step of RNA, introns are removed and the exons are ligated to form mRNA molecules [1]. Splicing is a complex multistep process catalyzed by a large, dynamic, multicomponent RNA–proteins complex called spliceosome that comprises U1, U2, U4, U5, and U6 small nuclear ribonucleoproteins (snRNPs) and many associated proteins [2]. In the human spliceosome, ~140 proteins are found to be associated with U snRNPs. Alternative pre-mRNA splicing produces more than many mRNA isoforms from a single gene, thus vastly expanding information contents of genes in eukaryotes [3]. In human, almost every gene yields more than one mRNA isoforms [4]. Aberrant alternative splicing is frequently found in human tumors [5,6]. Alternative splicing can result in malignancy by affecting expression levels of oncogenes and tumor suppressors [7,8]. In addition to other well-established hallmarks of cancer, altered splicing patterns have also been suggested as a new hallmark of cancer [9].

**Figure 1:**
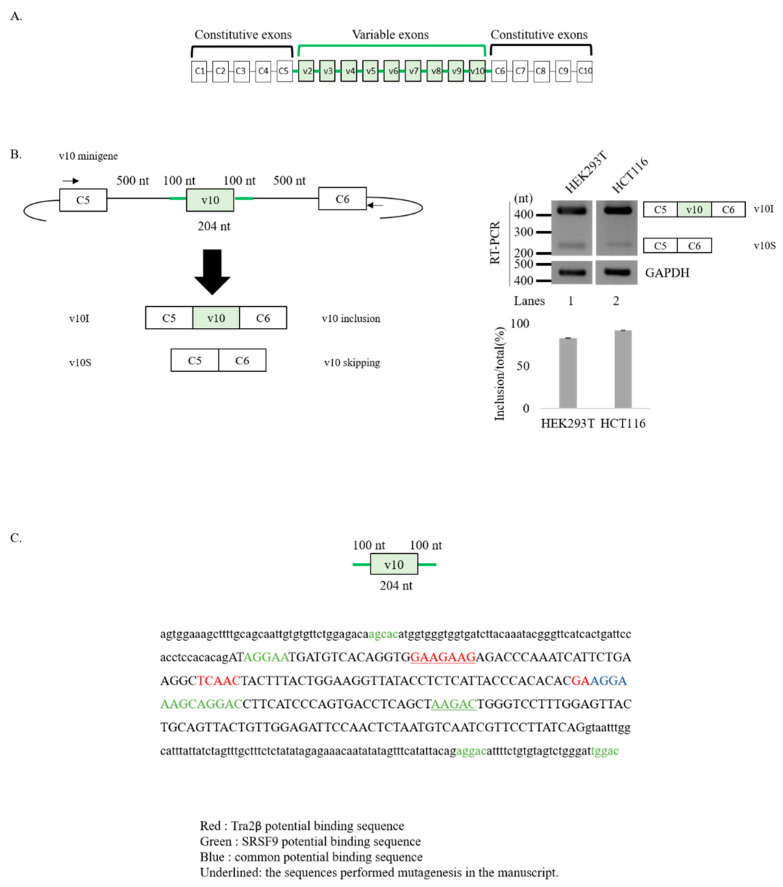
Representation of CD44 v10 minigene to study splicing of v10 exon. (**A**) Scheme of CD44 pre-mRNA. Exons are indicated by boxes. Introns are indicated by lines. Constitutive exons and introns are shown in black. Variable exons and introns are shown in green. (**B**) (Left) Schematic representation of v10 minigene is shown. v10 exon and its flanking introns are shown in green. Constitutive exons (C5 and C6) and flanking introns are shown in black. Length of each part is shown. Primers used in RT-PCR are indicated with arrows. (Right) Results of the RT-PCR analysis with RNAs extracted from HEK293T and HCT116 cells transfected with the v10 minigene are shown. Glyceraldehyde 3-phosphate dehydrogenase (GAPDH) is used as a loading control. Quantitation of v10 splicing is shown. Error bars indicate standard deviation (SD) from three independent experiments. (**C**) Potential binding motifs of Tra2β and SRSF9 identified by SpliceAid2 in CD44 v10 exon and flanking introns. RNA sequences in exon are shown in upper case. RNA sequences in introns are shown in lower case. Potential binding motifs of Tra2β are shown in red. The Tra2β binding cluster is underlined. Potential binding motifs of SRSF9 are shown in green. Overlapped binding sequences of Tra2β and SRSF9 are shown in blue. Sequences used to perform mutagenesis re underlined.

CD44 is a family of non-kinase transmembrane glycoproteins expressed in connective tissues, bone marrow, and embryonic stem cells [10]. Human CD44 has 19 exons, including 10 constitutive and nine variable exons [11] (Figure 1A). The standard CD44 form (CD44s) is encoded by constitutive exons. Variant CD44 form (CD44v) is produced by alternative splicing that comprise 10 constitutive exons and any combination of variable exons [11]. Thus, CD44v isoforms contain additional binding motifs to their ligands to promote interactions of CD44 with other molecules in the microenvironment [12]. Cancer cells undergoing epithelial to mesenchymal transition (EMT), one of the major mechanism by which cancer cells become metastatic [13], show an increased expression of CD44 as well as a switch from CD44s to CD44v [14,15]. A shift from CD44v to CD44s also occurred in the development of recurrent mesenchymal type of breast tumor in a murine model of breast cancer progression [15]. Orthotopic infection of CD44v8-10 isoform in breast cancer cells of mice can cause an increase in lung metastasis [16]. Increased CD44v can promote the invasive activity of endocrine-resistant breast cancer cells, while decreased CD44v can reduce the invasive capacity of such cells [16,17,18]. High expression of v10 exon of CD44 has been shown in malignant renal carcinoma cells [19]. It has been demonstrated that exon v10 promotes breast tumor progression and development [20]. In addition, v10 exon can promote leukocytes adhesion to bone marrow cells and migration to disease sites in mice [21,22,23]. Importantly, DNA aptamers specifically binding to v10 can inhibit the migration of breast cancer cells, indicating an exclusive role of v10 in cell migration and metastasis of breast cancer [24]. Despite the importance of v10 of CD44, the only splicing mechanism identified so far is from a study of our group showing that hnRNP L can regulate v10 splicing by targeting the upstream intron [25].

Alternative splicing is controlled by a large group of ubiquitously expressed splicing regulators characterized by the presence of an arginine–serine (RS)-rich domain (called SR proteins) [26]. Tra2β is a member of the SR protein family. It has an RNA recognition motif (RRM) flanked by RS domains at both sides [26,27]. Tra2 is known to control sexual differentiation and spermatogenesis of fruit flies [28,29]. Tra2 gene is duplicated in vertebrates to Tra2α and Tra2β [30,31]. Tra2β is known to promote the inclusion of a “poison” exon into the Tra2α mRNA, resulting in the insertion of an in-frame stop codon into the Tra2α mRNA to target it for nonsense mediated decay without allowing subsequent translation of Tra2α protein [30,32]. Tra2β can bind to AGAA and CAA RNA sequences to regulate alternative splicing [33,34]. Approximately 13% of recurrent mutations of Tra2β have been found in TCGA human breast tumors sorted by frequency [35]. Tra2β upregulation is enriched in basal breast tumors and an aggressive cancer subtypes. Indeed, increased Tra2β expression has been detected in ~40% of triple-negative breast cancers (TNBCs) [35]. SRSF9 is another member of SR protein family, which comprises RRMs and RS domains [26]. It has been demonstrated that SRSF9 can regulate cassette exon splicing, 5′ splice-site selection, and 3′ splice-site selection of the Bcl-x pre-mRNA [36,37,38]. SRSF9 can enhance β-catenin synthesis to promote Wnt-signaling-mediated tumorigenesis in multiple types of tumors [39].

In this study, we applied a minigene-based approach to determine the opposite roles of Tra2β and SRSF9 on CD44 v10 splicing. In addition, both proteins functionally target v10 exon, with Tra2β targeting the GAAGAAG sequence and SRSF9 targeting the AAGAC sequence located at CD44 exon v10. Thus, the ability of these RNA sequences to interact with Tra2β or SRSF9 proteins and the ability of these proteins to be the functional regulator of CD44 exon v10 splicing are well correlated. Collectively, our results provided new regulatory mechanisms for v10 exon of CD44.

## 2. Materials and Methods

### 2.1. Cell Culture, Transfection, RNA Extraction, and RT-PCR

HEK293T and HCT116 cells were grown in a 5% CO_2_ incubator at 37 °C in Dulbecco’s Modified Eagle Medium (DMEM) and Roswell Park Memorial Institute Medium (RPMI) medium containing 10% fetal bovine serum (FBS, HyClone, Logan, UT, USA), 2 mM glutamine, 100 iU/mL penicillin, and 100 μg streptomycin, as described previously [25]. Polyethyleneimine (PEI) (Sigma-Aldrich, Saint Louis, MO, USA) was used to transfect CD44 v10 minigenes and expression vectors of Tra2β or SRSF9 into HEK293T and HCT116 cells [40]. An amount of 1.0 μg plasmid DNAs were mixed with 2.0 μg PEI reagent in 100 μL media and incubated at room temperature for 20 min before adding to culture plate. Total RNAs were extracted at 48 h after transfection using RiboEX reagent (GeneAll, Seoul, Korea) according to the manufacturer’s instructions. Reverse transcription was performed using Moloney Murine Leukemia Virus (M-MLV) reverse transcriptase (ELPIS, Daejeon, Korea) with 1 μg RNA and oligo-dT18 primer. Primer sequences used in this study are listed in Table S1.

### 2.2. Plasmid Construction

CD44 v10 minigenes were described previously [25], whereas its mutated versions (M-Tra2β and M-SRSF9 minigenes) were produced by overlapping PCR using primers (T_F_/T_R_ and S_F_/S_R_). All primer sequences used to generate these plasmids are listed in Table S1.

### 2.3. RNA Immunoprecipitation Assay

RNA pulldown analysis was performed, as described previously [25]. Briefly, streptavidin agarose was used to covalently link 5′-end biotin-labeled RNA by incubating with buffer D (20 mM Tris-Cl pH 7.5, 150 mM KCl, 0.2 mM EDTA, 10% glycerol, 0.5 mM DTT, 0.5 mM PMSF) at 4 °C for 1h. RNA-linked streptavidin beads were incubated with cell lysates for 4 h at 4 °C and then washed with buffer D five times. Beads were loaded onto 10% SDS-PAGE gel and subjected to immunoblotting analysis using anti-Tra2β (Santa Cruz, sc-166829, Dallas, TX, USA) and anti-SRSF9 (Santa Cruz, sc-134036) antibodies. Biotin-labeled RNA sequences are shown in Table S1.

### 2.4. Immunoblotting Assay

Cells were incubated with cell lysis buffer (0.1% triton X-100, 50 mM Tris-Cl, pH 7.5, 150 mM NaCl, 5 mM EDTA, 1 mM beta-mercaptoethanol) at 4 °C for 30 min. Proteins were separated with 12% SDS-PAGE gel followed by transferring to nitrocellulose membrane. Tra2β, SRSF9, and α-tubulin were detected using anti-Tra2β (Abcam, ab31353, Cambridge, UK), anti-SRSF9 (Santa Cruz, sc-134036), and anti-α-tubulin (Abcam, ab18251) antibodies, respectively.

### 2.5. RNA-Binding Motifs Prediction

Tra2β putative RNA-binding motifs prediction was performed using SpliceAid 2 (http://www.introni.it/spliceaid.html) [41]. Human CD44 v10 exon with 5′ and 3′ 100 nucleotide sequences were analyzed for human/mouse motifs. To confirm the identified motifs, the same region was analyzed using RBPmap web server (http://rbpmap.technion.ac.il) [42], with the following parameters: stringency level = low. In Table S2, only splicing factor frequently altered in breast cancers (>5% of tumor specimens) are shown. Predicted motifs by hnRNP L, a known regulator of CD44 v10 exon [25], are shown as controls.

### 2.6. Quantitation and Statistical Analyses

RT-PCR, immunoblotting, and immunoprecipitation experiments were performed in triplicate. RT-PCR products were loaded into agarose gels followed by staining with Ethidium Bromide (EtBr). Image J program was used to quantify the bands. Data are presented as means ± SD (standard deviation of the mean). Statistical differences between three groups were determined using one-way ANOVA. Statistical significance was marked as **** *p* < 0.0001, *** *p* < 0.001, ** *p* < 0.01, and * *p* < 0.05.

## 3. Results

### 3.1. v10 Exon Contains Binding Motifs of Tra2β and SRSF9

As shown in Figure 1A, CD44 pre-mRNA comprises nine variable exons (v2–v10) that are included in the mature mRNA in a very complicated combinations [43,44]. Thus, it is hard to study splicing regulatory mechanisms of the endogenous CD44 pre-mRNA. For this reason, we applied a minigene assay using a plasmid containing only v10 exon (204 nucleotides (nt)), its flanking upstream (100 nt) and downstream introns (100 nt), constitutive C5 exon and its downstream intron (500 nt), and constitutive C6 exon and its upstream intron (500 nt) [25] (Figure 1B, left). This v10 minigene was expected to produce an mRNA isoform in which v10 exon is included (v10I), and an mRNA transcript in which v10 exon is skipped (v10S) (Figure 1B). v10I isoform represents v10 exon spliced mRNA, whereas v10S isoform represents v10 exon unspliced mRNA. To specifically detect only minigene transcripts, we performed an RT-PCR analysis annealing with the vector sequence and another primer targeting C5 exon (Figure 1B). Consistent with previous results [25], the minigene produced v10I isoform dominantly in both HEK293T (~83%, lane 1) and HCT116 (~92%, lane 2) independently (Figure 1B, right), indicating that v10 exon was efficiently spliced. Small amounts of v10 unspliced v10S were also produced in HEK293T and HCT116. Thus, we used this minigene to identify regulatory mechanisms of v10 exon splicing.

We have previously demonstrated that hnRNP L inhibits CD44 v10 splicing by binding an evolutionally conserved CA-rich sequence within intron 9 [25]. In order to identify additional splicing RNA regulators, we analyzed CD44 v10 exon sequence and the adjacent intronic regions (100 nt) with the SpliceAid2 (http://www.introni.it/spliceaid) [41] and RBPmap (http://rbpmap.technion.ac.il) [42] bioinformatic tools (Table S2). Considering the importance of CD44 splicing for cancer progression, among the different RNA binding proteins (RBP) with predicted binding motifs in CD44 v10 region, we decided to focus our attention on those proteins whose expression levels are frequently altered in different cancer types [35]. In particular, the exonic GAAGAAG motif (Figure 1C) represents a predicted binding site for Tra2β in both bioinformatics analyses (Figure 1C, Table S2). In addition to this cluster, SpliceAid2 was able to predict another two Tra2β binding motifs in CD44 exon v10 (red, Figure 1C; Table S2). Remarkably, Tra2β has recently been found altered in approximately 13% of breast cancer specimens [35], 0.5–1.5% of hematological malignancies [7], and in lung cancers [45]. Additionally, among splicing regulators frequently altered in breast cancers (in >5% of tumors [35]), we found different members of SR protein family (such as SRSF1, SRSF2, SRSF3, SRSF4, SRSF6, SRSF9, SRSF10, SRSF11) with at least one predicted binding motif in the CD44 v10 region (Table S2). Among these, the highest number of predicted motifs were for SRSF9—previously known as SRp30c—(AGCAC, AGGAA, AGCAG, AGGAC, AAGAC, and UGGAC). In particular, by using SpliceAid2, we found eight potential SRSF9 binding motif: one in intron 9, four in exon v10, and two in intron 10 (green). Interestingly, one RNA-element was predicted to interact with both Tra2β and SRSF9 (blue). The presence of potential binding sequences for Tra2β and SRSF9 provide the rationale to further investigation of their function in CD44 v10 splicing.

### 3.2. Tra2β and SRSF9 Regulate CD44 v10 Exon Splicing in Opposite Directions

**Figure 2:**
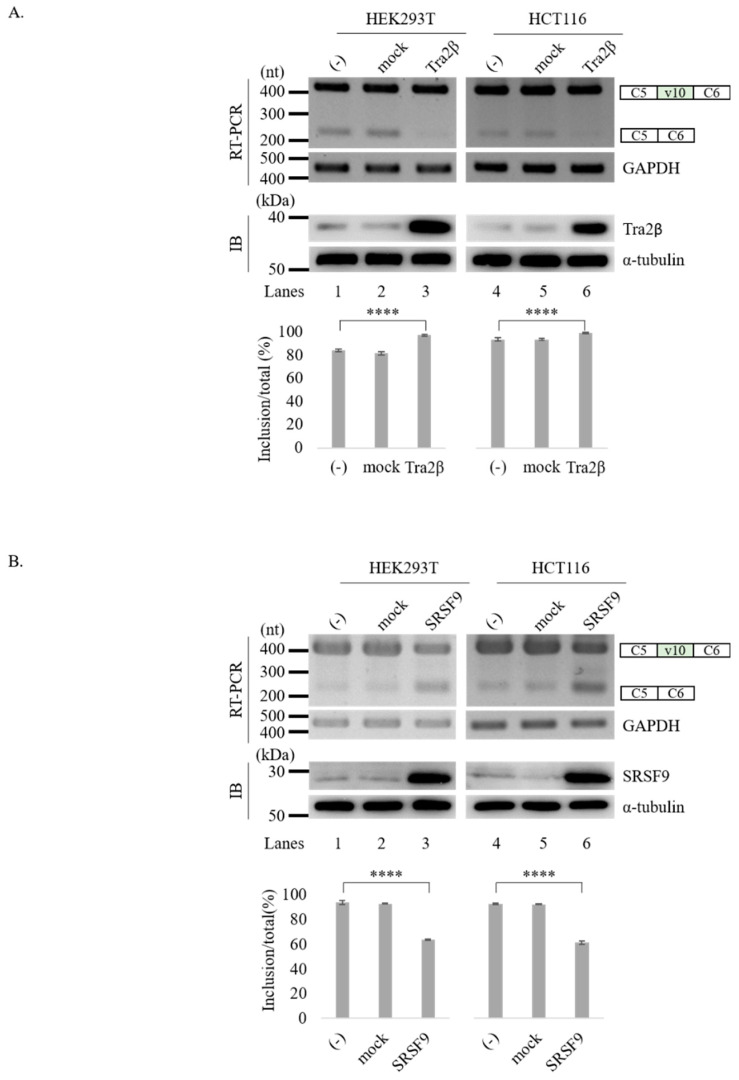
Tra2β and SRSF9 regulate v10 exon splicing with opposite directions. (**A**) (Upper) RT-PCR analysis of RNAs from HEK293T (left) and HCT116 (right) cells co-transfected with v10 minigene and the Tra2β expressing vector. GAPDH is shown with a loading control. Immunoblotting of Tra2β and α-tubulin expression levels are shown. Whole blots of western blot analysis for Figure 2A are shown in Figure S2. (Lower) Statistical analysis graphs of RT-PCR with *p* values. **** *p* < 0.0001. (**B**) (Upper) RT-PCR analysis of RNAs from HEK293T (left) and HCT116 (right) cells transfected with v10 minigene and SRSF9 expression vector. GADPH is shown with a loading control. Immunoblotting of SRSF9 and α-tubulin expression levels are shown. Whole blots of Western blot analysis for Figure 2B are shown in Figure S2. (Lower) Statistical analysis of RT-PCR results with *p* values. **** *p* < 0.0001.

To test the possibility that Tra2β and SRSF9 could regulate CD44 v10 splicing, we co-transfected the CD44 v10 minigene and vectors over-expressing Tra2β and SRSF9 proteins into HEK293T and HCT116 cells. In accord with our bioinformatic analysis, we found that Tra2β promoted v10 exon inclusion significantly in both HEK293T (~13%, lane 3) and HCT116 cells (~6%, lane 6) and inhibited v10 skipping, accordingly. Opposite to the function of Tra2β, we found that SRSF9 inhibited v10 exon inclusion significantly in both HEK293T (~30%, lane 3) and HCT116 cells (~31%, lane 6). However, in both experiments, control plasmid did not affect v10 exon splicing (Figure 2A,B). These results indicate that Tra2β and SRSF9 regulate CD44 v10 exon splicing with opposite effects on v10 exon inclusion. We further wondered whether reduced expression of Tra2β and SRSF9 could affect splicing of upstream and downstream intron of v10. As shown in Figure S1, shRNA treatment of neither Tra2β nor SRSF9 could affect flanking intron splicing of v10 (Figure S1B, lanes 3 and 4), indicating that reduced expression of Tra2β and SRSF9 did not affect splicing of endogenous flanking introns of v10. We next asked if knockdown (KD) of Tra2β and SRSF9 in the cells that endogenously overexpress Tra2β and SRSF9. From the Broad Institute of Cancer Cell Line Encyclopedia (CCLE) (https://portals.broadinstitute.org/ccle), we found that HCT116 cells express high endogenous Tra2β and SRSF9 (Table S3). Similar to the results observed in HEK293T cells, we were not able to observe the KD effects of these two genes on endogenous CD44 v10 splicing (Figure S1C). These results indicate that there are more complicated control mechanisms in the endogenous CD44 v10 splicing, as it forms diverse mRNA isoforms with other various exons.

### 3.3. Tra2β Functionally Targets GAAGAAG Sequence in v10 Exon to Regulate Splicing of v10 Exon

**Figure 3:**
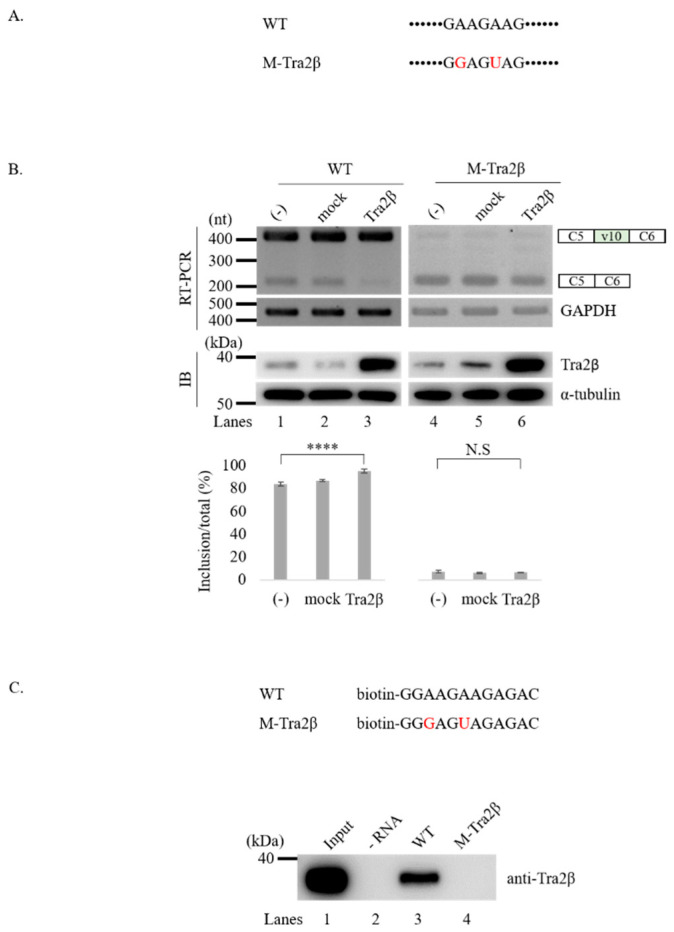
Tra2β functionally targets GAAGAAG sequence in CD44 v10 exon to regulate its inclusion. (**A**) Potential Tra2β binding sequence and its mutated versions in CD44 v10 minigene called M-Tra2β vector are shown. Mutated nucleotides are shown in red. (**B**) (Upper) RT-PCR analysis of RNAs from HEK293T cells transfected with wild-type and M-Tra2β minigene and Tra2β over-expression plasmid. GADPH is shown with a loading control. Immunoblotting of Tra2β and α-tubulin expression levels are shown. Whole blots of Western blot analysis for Figure 3B are shown in Figure S3. (Lower) Statistical analysis of RT-PCR results with *p* values. **** *p* < 0.0001. (**C**) (Upper) Biotin-labeled oligonucleotides of potential Tra2β and its mutant binding sites are shown. Whole blots of Western blot analysis for Figure 3C are shown in Figure S3. (Lower) RNA pulldown analysis with wild-type, M-Tra2β oligos.

Because Tra2β is an RBP, we next sought to determine functional target sequences in CD44 v10 exon or its flanking introns. Among the predicted Tra2β binding motifs (Figure 1C), we focused on the GAAGAAG cluster sequence located at 22nt downstream from the 3′ splice-site of v10 exon, as it was (i) located closer to the 3′ splice-site of v10 exon; (ii) best ranked (Z-score: 3.304; *p*-value: 4.77 × 10^−4^) in RBPmap prediction; (iii) identified by both SpliceAid2 and RBPmap tools. To test the possibility that Tra2β could affect v10 splicing through interacting with this sequence, we mutated the GAAGAAG (called wild-type) sequence into GGAGUAG (called M-Tra2β) sequence in the CD44 v10 minigene (Figure 3A). If this sequence is the functional target of Tra2β protein, M-Tra2β minigene should show reduced v10 inclusion compared to the wild-type minigene upon Tra2β over-expression. As shown in Figure 3B, v10 inclusion was significantly reduced in the M-Tra2β minigene (lanes 4, ~7%), and importantly, this effect was also observed after increased Tra2β expression levels (lanes 6). Collectively, this analysis suggests that Tra2β could potentially functions through the GAAGAAG sequence to regulate CD44 v10 splicing.

We next wondered the relationship between binding ability of RNA motifs and its promoting activity of v10 exon. To analyze physical interaction between Tra2β and GAAGAAG, we synthesized biotin-labeled wild-type and M-Tra2β RNA to perform an RNA-pulldown assay using streptavidin followed by immunoblotting with anti-Tra2β antibody (Figure 3C, upper panel). As shown in Figure 3C (lower panel), Tra2β strongly interacted with the wild-type binding motif in v10 exon (lanes 3), whereas it was not able to interact with the mutated M-Tra2β RNA (lanes 4). Therefore, we could conclude that the ability of Tra2β to interact with GAAGAAG RNA motif is correlated with its ability to promote v10 inclusion.

### 3.4. SRSF9 Targets AAGAC Sequence in v10 Exon to Inhibit v10 Exon Inclusion

**Figure 4:**
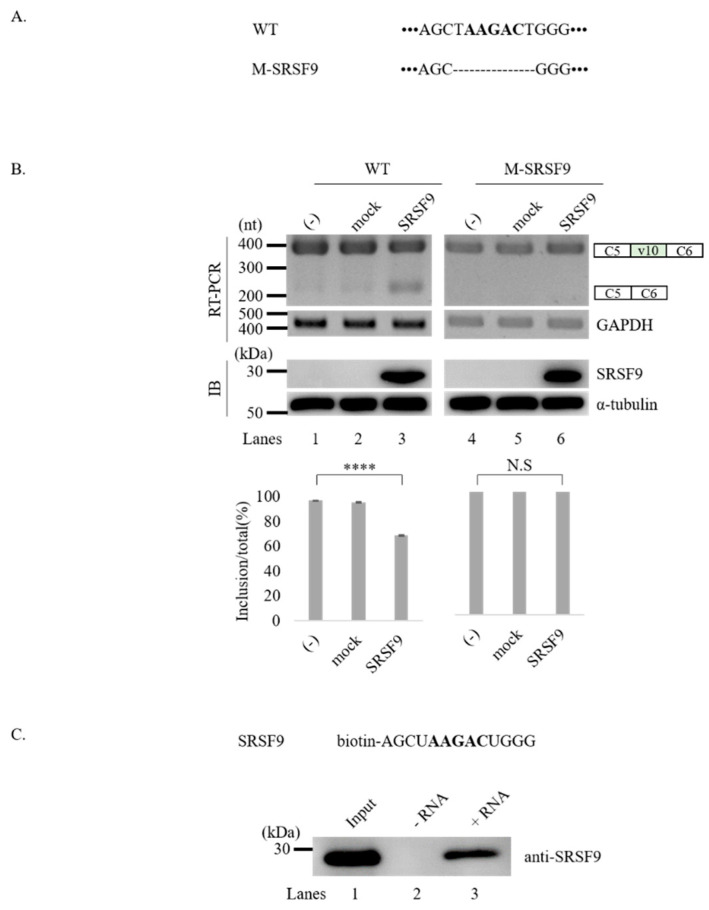
SRSF9 targets AAGAC sequence in v10 exon to inhibit its inclusion. (**A**) Potential SRSF9 binding sequence and mutated sequence in M-SRSF9 minigene are shown. (**B**) (Upper) RT-PCR analysis of RNAs from HEK293T cells transfected with wild-type and M-SRSF9 v10 minigenes along with SRSF9 expression plasmid. GADPH is shown as a loading control. Immunoblotting of SRSF9 and α-tubulin expression levels are shown. Whole blots of Western blot analysis for Figure 4B are shown in Figure S3. (Lower) Statistical analysis of RT-PCR results with *p* values. **** *p* < 0.0001. (**C**) (Upper) Biotin-labeled oligonucleotides of potential SRSF9 binding sites are shown. Whole blots of Western blot analysis for Figure 4C are shown in Figure S3. (Lower) RNA pulldown analysis with SRSF9 oligos.

We next asked whether SRSF9 could also target the regulatory function of v10 splicing. From eight potential binding motifs shown in Figure 1C, we focused on the AAGAC sequence (green, underlined) located at 132nt downstream from the 3′ splice-site of v10 exon. To test the possibility that the AAGAC sequence could function as the target of SRSF9, we produced a CD44 v10 minigene in which this sequence was deleted (M-SRSF9) (Figure 4A). Since SRSF9 inhibited v10 inclusion (Figure 2B), we expected that M-SRSF9 minigene would have more v10 inclusion compared to wild-type minigene. Because wild-type v10 minigene produced v10 included isoform dominantly, increased v10 included isoform was hard to detect. Interestingly, we found that M-SRSF9 produced exclusively the v10 included isoform (Figure 4B, lanes 4) (~100%). We further expected that SRSF9 function on v10 exon splicing would be abolished in M-SRSF9 minigene. As predicted, we found that SRSF9 overexpression did not have an inhibitory role on splicing of the transcripts generated from the M-SRSF9 minigene (lanes 6). These results indicate that the AAGAC sequence is the functional targets of SRSF9 in CD44 v10 exon splicing. We next asked whether SRSF9 could interact with the AAGAC sequence. To this aim, we performed RNA-pulldown assay using biotin-labeled SRSF9 binding oligo comprising AAGAC and flanking four oligonucleotides (Figure 4C, upper). As shown in Figure 4C (lower panel), SRSF9 was able to be associated with the AAGAC sequence (lanes 3), whereas no interaction was observed without adding biotin-labeled RNA (lanes 2). These results indicate that that AAGAC is the functional target of SRSF9 through which this RBP could regulate CD44 exon v10 splicing.

## 4. Discussion

Different from regular skipped exons, which include only one alternative exon, human CD44 pre-mRNA contains 10 various exons instead. Thus, CD44 pre-mRNA is able to produce ~10^3^ kinds of various mRNA alternative splicing isoforms [43]. In the present study, regulatory splicing mechanisms of CD44 v10 exon (encoding for a region in the extracellular domain of the protein) were investigated. By specifically focusing on v10, we applied a minigene that only included v10 alternative splicing exon, not other various exons. The v10 exon and its flanking introns are inserted between constitutive C5 and C6 exons with their flanking introns. Using this minigene, we were able to identify Tra2β and SRSF9 as regulatory proteins of v10 exon definition. We found that Tra2β and SRSF9 affected v10 splicing in opposite directions. While Tra2β promoted v10 inclusion, SRSF9 inhibited its inclusion. These proteins targeted sequences in v10 exon to regulate v10 splicing, with Tra2β targeting GAAGAAG and SRSF9 targeting AAGAC. Importantly, Tra2β and SRSF9 physically interacted with these sequences. Therefore, our results suggest that Tra2β and SRSF9 can directly interact with v10 exon to regulate v10 splicing in opposite directions.

Splicing regulatory mechanisms of various exon other than v10 in CD44 have been studied previously [25,44,46,47]. For example, we have demonstrated that SRSF2 and hnRNP A1 regulate splicing of v6 exon [44]. Based on the analysis of The Cancer Genome Atlas (TCGA), v10 is one of the most abundant variable exons in CD44, and its expression has been shown to be most highly correlated among the breast cancer specimens [48]. The only splicing regulatory factor identified to be involved in v10 splicing is hnRNP L [25]. Here we found two new regulatory proteins: Tra2β and SRSF9 with opposite roles, both targeting v10 exon. Oncogenic activity of Tra2β and SRSF9 has been demonstrated previously [30,35,36]. How their effects on v10 exon splicing of CD44 links their oncogenic functions need to be determined. Our findings provide a novel insight for the regulation of v10 exon in CD44 pre-mRNA. We cannot exclude that the possibility that additional splicing factors are involved in the tight control of v10 exon splicing by directly promoting or repressing exon recognition, or by indirectly preventing or favoring the recruitment of hnRNPL, Tra2β, and SRSF9. Nevertheless, our prediction of RNA-binding motifs in v10 exon and adjacent introns provides a valuable tool for the identification of additional regulatory elements.

We were not able to detect the effects of Tra2β and SRSF9 on endogenous v10 splicing. shRNA effects are not always opposite to the overexpression results. The difference might be caused by the differences of experimental designs: overexpression or shRNA, minigene, or endogenous. It is also possible that, in the minigene, v10 exon is linked to C5 and C6 exons, while endogenous v10 exon is linked to v9 and C6. Another possibility is that other various exons also play important roles in the v10 selection by overexpression or shRNA. How these exons function each other needs to be determined.

## 5. Conclusions

Tra2β and SRSF9 regulate alternative CD44 v10 splicing through interacting with GAAGAAG and AAGAC independently.

## Supplementary Materials

The following are available online at https://www.mdpi.com/2072-6694/12/11/3195/s1, Table S1: List of Primers used for RT-PCR and RT-Qpcr, Table S2: List of predicted RNA-binding motifs in CD44 v10 exon and flanking introns using SpliceAid2 and RBPmap, Table S3: Expression of Tra2β and SRSF9 in different cancer cells identified from the Broad Institute of Cancer Cell Line Encyclopedia (CCLE), Figure S1: (A) Primers used in RT-PCR are shown with arrows and numbers. (B–C) RT-PCR analysis of endogenous v10 splicing with RNAs from HEK293T (B) and HCT116 (C) cells treated with shRNAs targeting SRSF9 or Tra2β(lanes 3 and 4). RT-PCR from untreated or non-silencing shRNA treated cells were used as controls (lanes 1 and 2). RT-PCR analysis of SRSF9 or Tra2β RNAs are also shown. Figure S2: Whole blots of Western blotting for Figure 2A,B. Figure S3: Whole blots of Western blotting for Figure 3B,C. Figure S4: Whole blots of Western blotting for Figure 4B,C.
