# Supplementary material for: Opposite Roles of Tra2β and SRSF9 in the v10 Exon Splicing of CD44
Source: Cancers (Basel). 2020 Oct 30;12(11):3195. doi: 10.3390/cancers12113195 (PMC7692347; doi:10.3390/cancers12113195)
Supplement: Supplementary file 1 [file cancers-12-03195-s001.zip › Supplementary figures.pdf]

Supplementary Figure 1.

A.

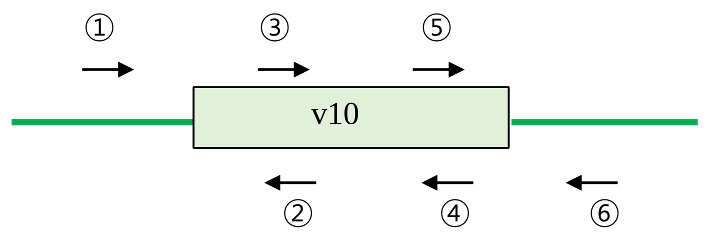

B. HEK293T

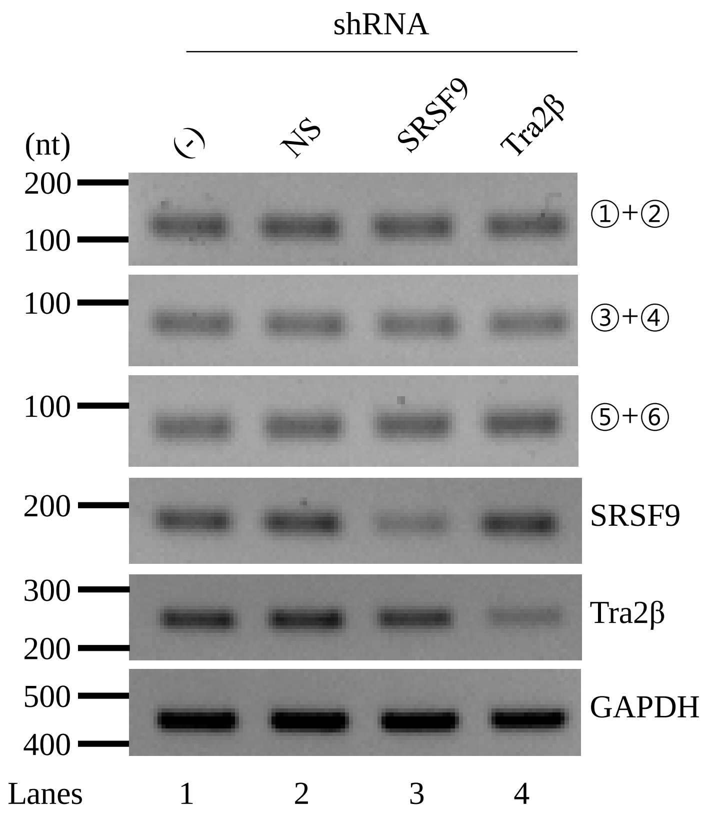

C. HCT116

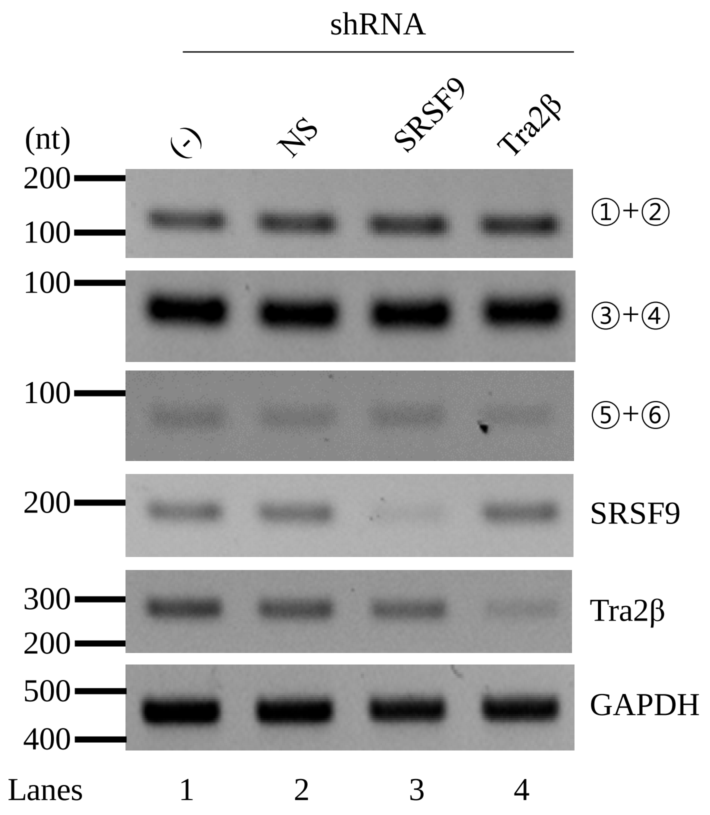

Supplementary Figure 2.

<Fig.2>

(A) - Tra2β

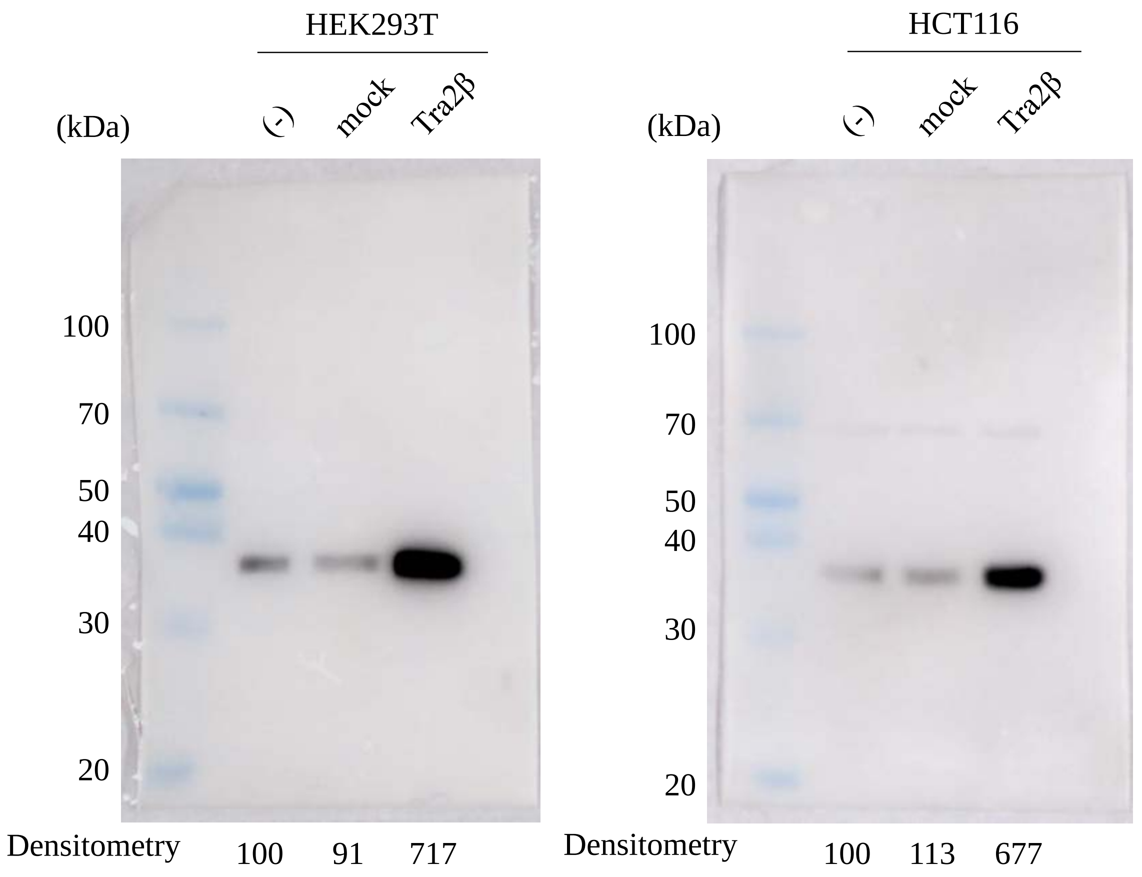

(A) - α-tubulin

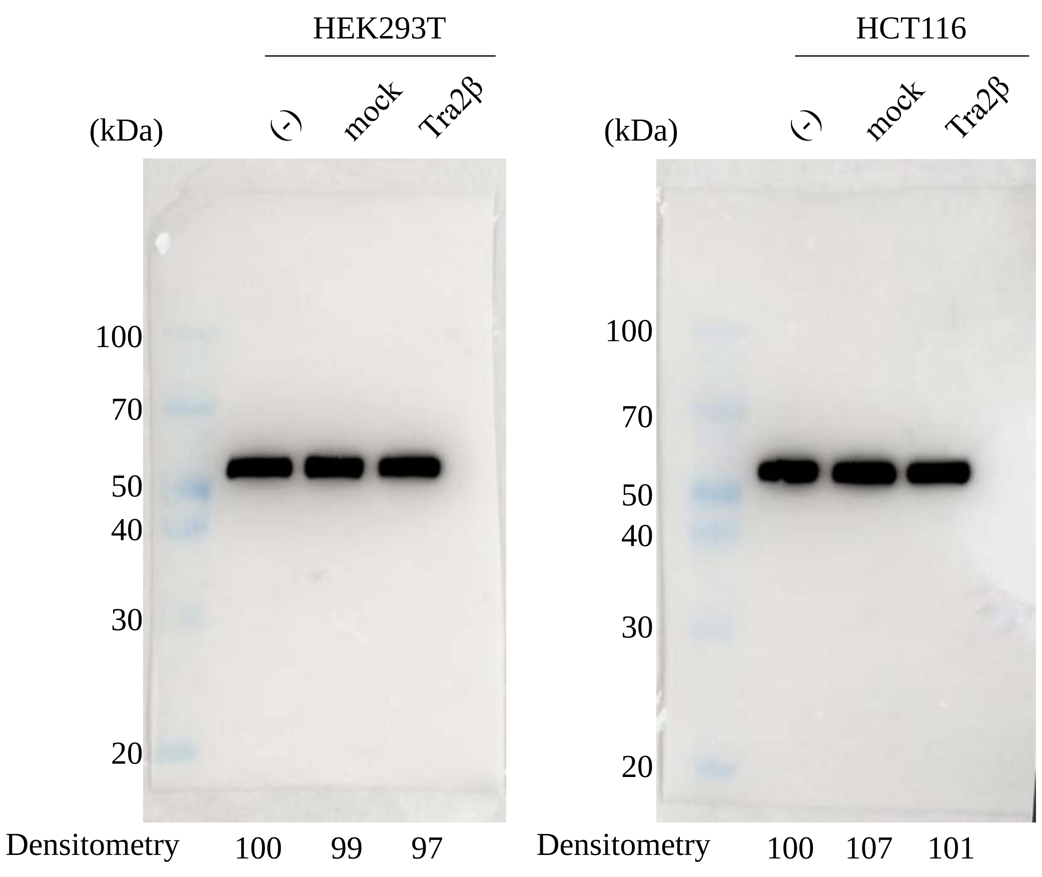

(B) – SRSF9

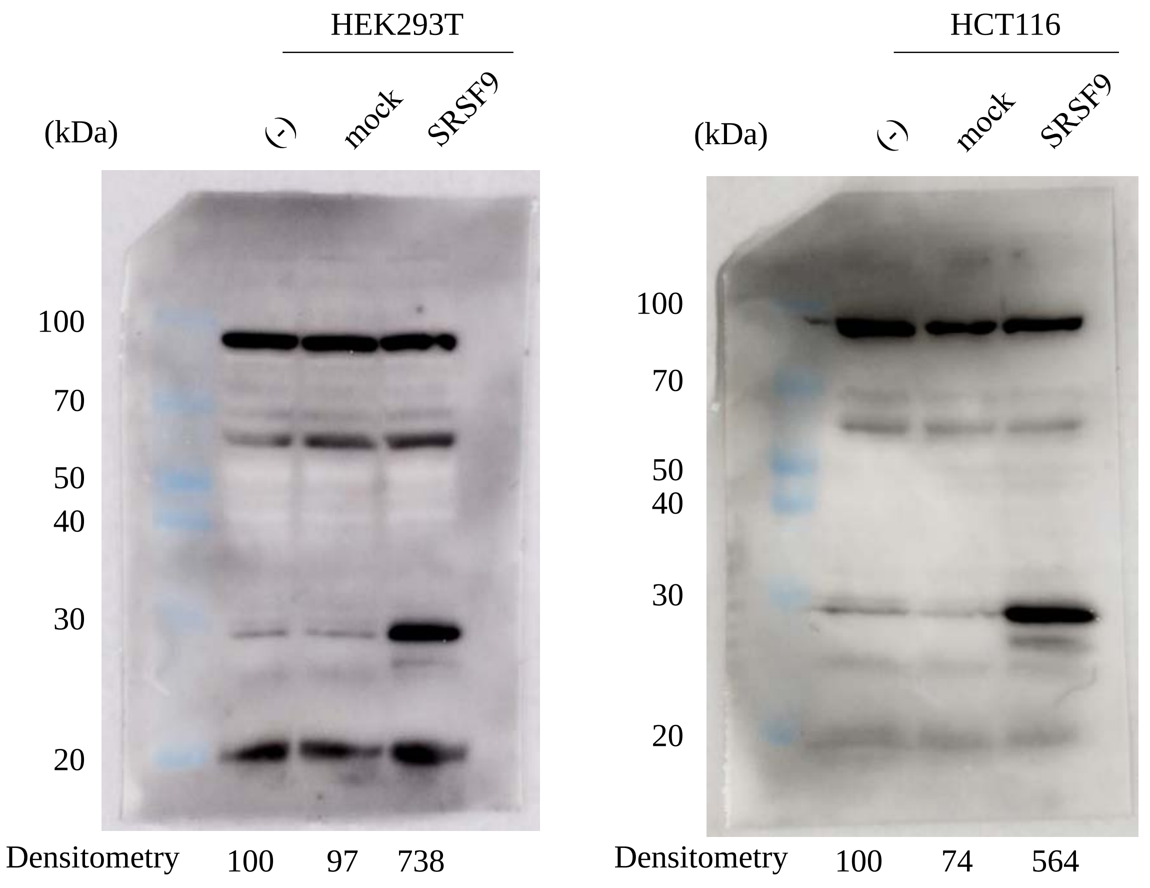

(B) - α-tubulin

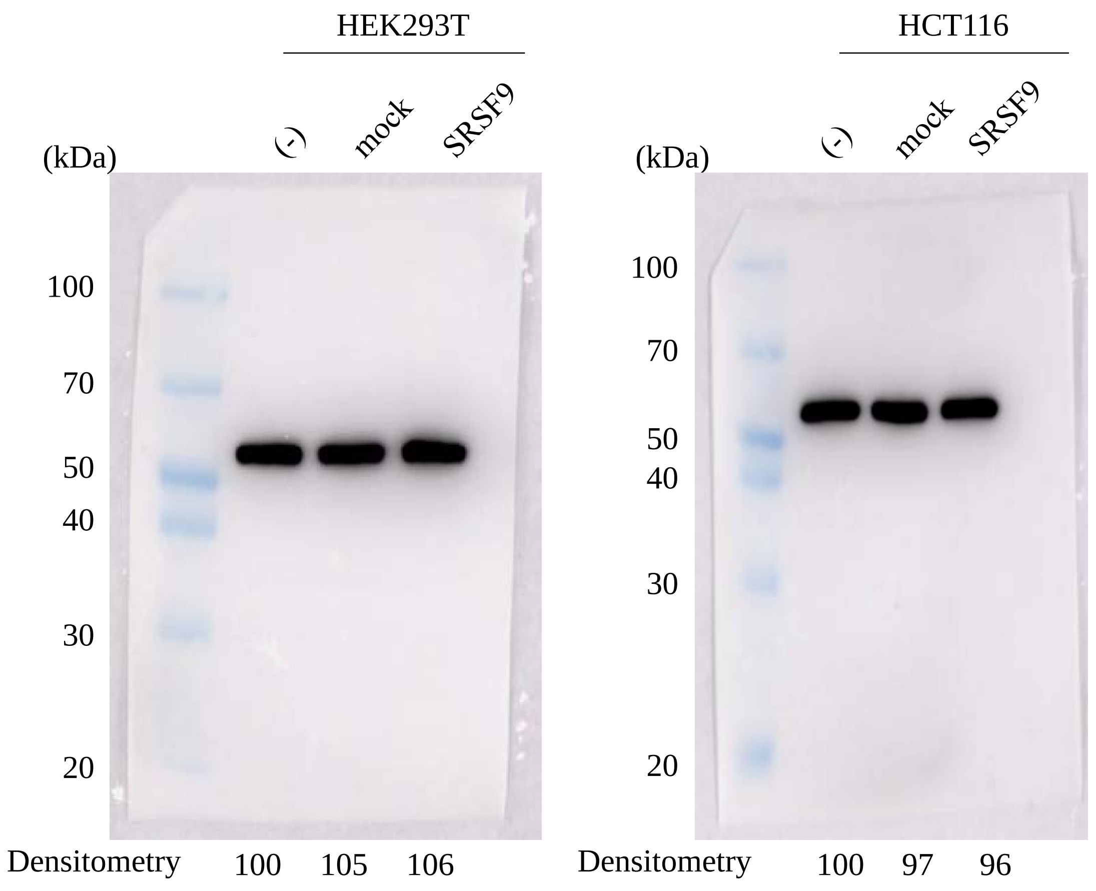

Supplementary Figure 3.

<Fig.3>

(B) - Tra2β

WT: (-) mock Tra2β (kDa)

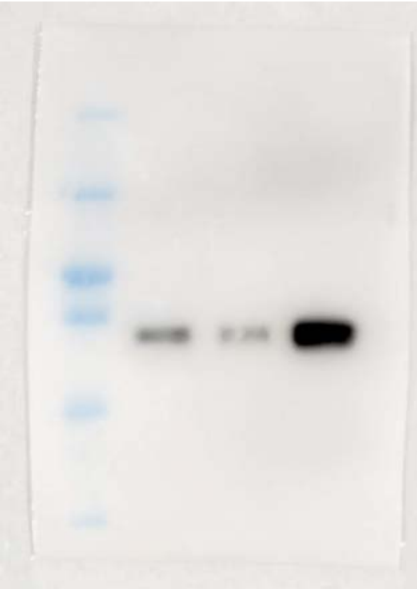

100 70 50 40 30 20

Densitometry 100 74 534

M-Tra2β: (-) mock Tra2β (kDa)

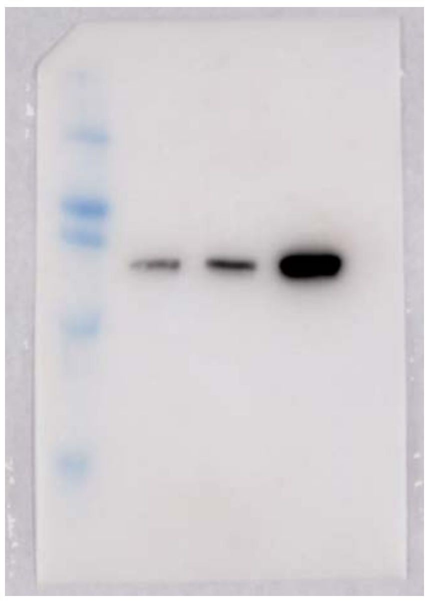

70 50 40 30 20

Densitometry 100 121 564

(B) - α-tubulin

WT: (-) mock Tra2β (kDa)

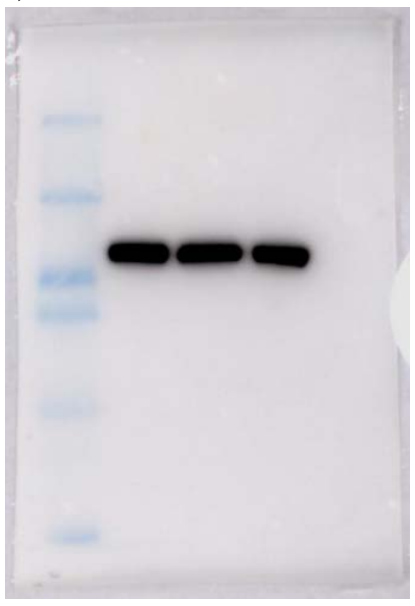

100 70 50 40 30 20

Densitometry 100 105 97

M-Tra2β: (-) mock Tra2β (kDa)

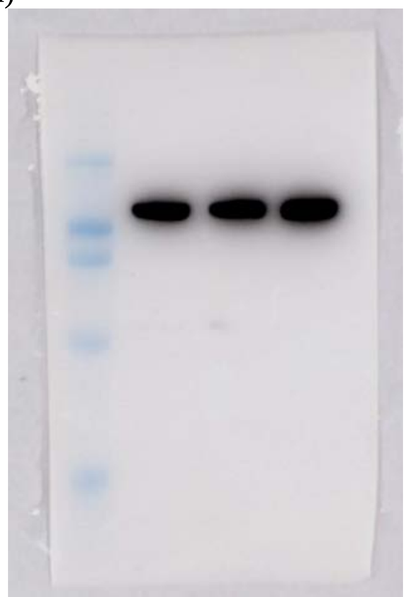

70 50 40 30 20

Densitometry 100 100 105

(C)

(kDa) Input -RNA WT M-Tra2β

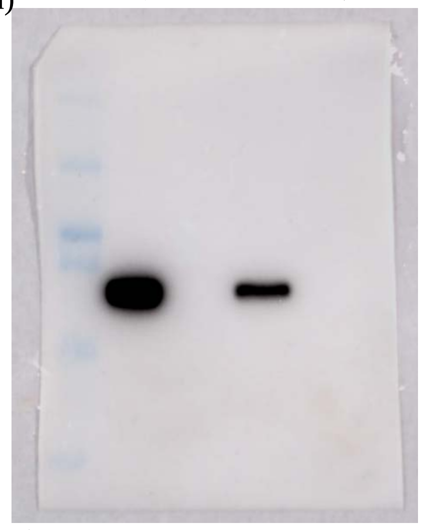

100 70 50 40 30 20

Densitometry 100 0 34 0

<Fig.4>

(B) – SRSF9

WT: (-) mock SRSF9 (kDa)

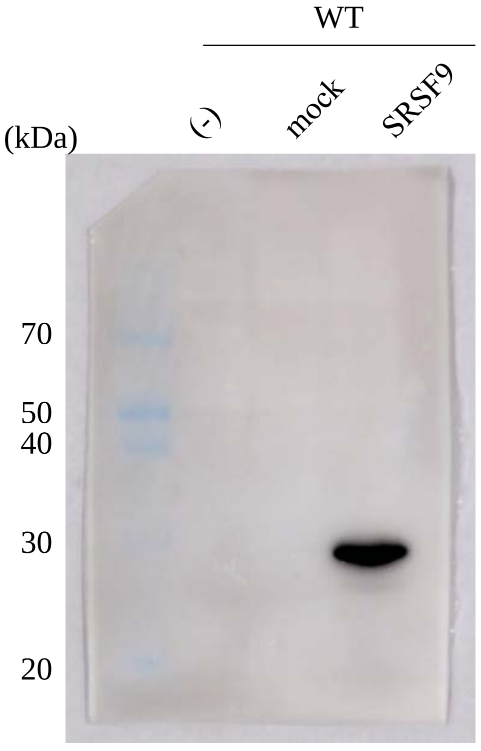

70 50 40 30 20

Densitometry 0 0 100

M-SRSF9: (-) mock SRSF9 (kDa)

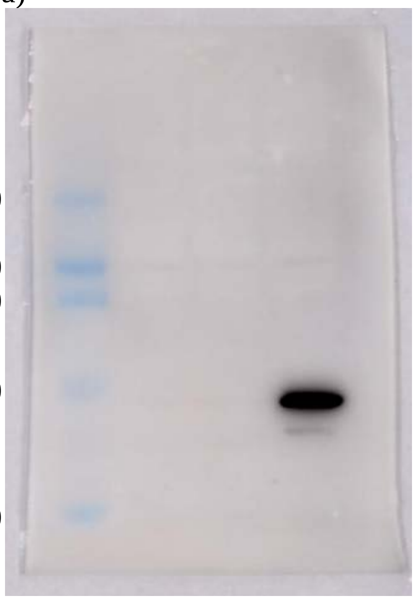

70 50 40 30 20

Densitometry 0 0 100

(B) - α-tubulin

WT: (-) mock SRSF9 (kDa)

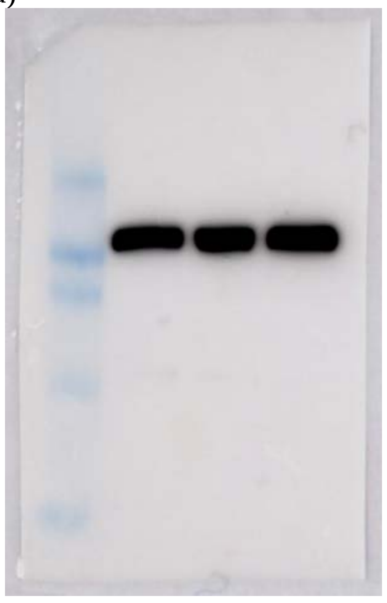

70 50 40 30 20

Densitometry 100 95 106

M-SRSF9: (-) mock SRSF9 (kDa)

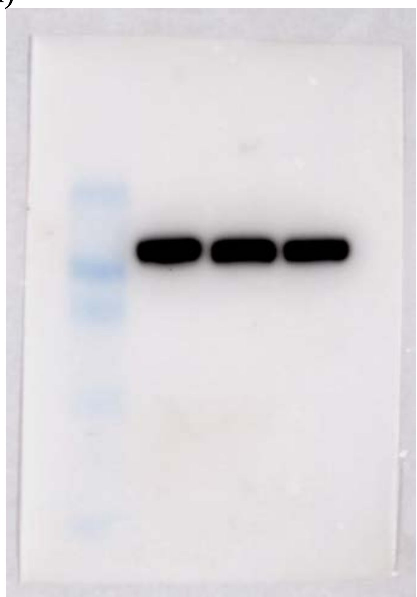

70 50 40 30 20

Densitometry 100 101 96

(C)

(kDa) Input -RNA +RNA

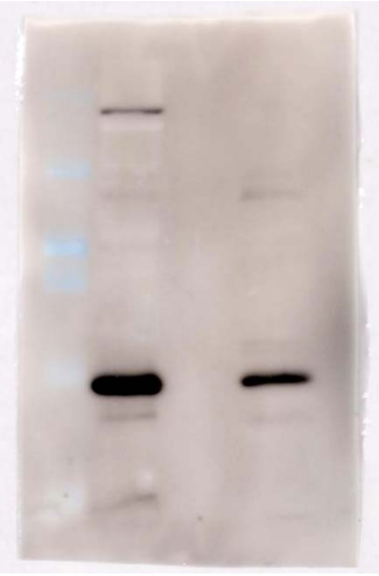

100 70 50 40 30 20

Densitometry 100 0 36
